# *SNORKEL* Genes Relating to Flood Tolerance Were Pseudogenized in Normal Cultivated Rice

**DOI:** 10.3390/plants11030376

**Published:** 2022-01-29

**Authors:** Keisuke Nagai, Yusuke Kurokawa, Yoshinao Mori, Anzu Minami, Stefan Reuscher, Jianzhong Wu, Takashi Matsumoto, Motoyuki Ashikari

**Affiliations:** 1 Bioscience and Biotechnology Center, Nagoya University, Nagoya 464-8601, Japan; kuroyu@meijo-u.ac.jp (Y.K.); nattouyo@gmail.com (Y.M.); anzu.minami@riken.jp (A.M.); reuscher.stefan@gmail.com (S.R.); ashi@agr.nagoya-u.ac.jp (M.A.); 2 Bioproductivity Informatics Research Team, RIKEN Center for Sustainable Resource Science, 1-7-22 Suehiro-cho, Tsurumi, Yokohama 230-0045, Japan; 3 Kihara Institute for Biological Research, Yokohama City University, 641-12 Maioka-cho, Totsuka, Yokohama 244-0813, Japan; 4 Institute of Crop Science, NARO, Tsukuba 305-8602, Japan; jzwu@affrc.go.jp (J.W.); tm206170@nodai.ac.jp (T.M.)

**Keywords:** submergence tolerance, APETALA2/ethylene-responsive factor, internode elongation, gene duplication, pseudogene

## Abstract

*SNORKEL1* (*SK1*) and *SNORKEL2* (*SK2*) are ethylene responsive factors that regulate the internode elongation of deepwater rice in response to submergence. We previously reported that normal cultivated rice lacks *SK* genes because the Chromosome 12 region containing *SK* genes was deleted from its genome. However, no study has analyzed how the genome defect occurred in that region by comparing normal cultivated rice and deepwater rice. In this study, comparison of the sequence of the end of Chromosome 12, which contains *SK* genes, between normal and deepwater rice showed that complicated genome changes such as insertions, deletions, inversions, substitutions, and translocation occurred frequently in this region. In addition to *SK1* and *SK2* of deepwater rice, gene prediction analysis identified four genes containing AP2/ERF domains in normal cultivated rice and six in deepwater rice; we called these genes *SK-LIKE* (*SKL*) genes. *SK*s and *SKL*s were present in close proximity to each other, and the *SKL*s in normal cultivated rice were in tandem. These predicted genes belong to the same AP2/ERF subfamily and were separated into four types: SK1, SK2, SKL3, and SKL4. Sequence comparison indicated that normal cultivated rice possesses a gene with high homology to *SK2*, which we named *SKL1*. However, none of the predicted *SKL*s except for *SKL3*s were expressed during submergence. Although *SKL3*s were expressed in both normal and deepwater rice, normal rice does not undergo internode elongation, suggesting that its expression does not contribute to internode elongation. Plants overexpressing *SKL1*, which showed the most homology to *SK2*, underwent internode elongation similar to plants overexpressing *SK1* and *SK2* under normal growth conditions. A yeast one-hybrid assay showed that the C-end of SKL1 has transcription activity, as do the C-ends of SK1 and SK2. Our results suggested that *SKL*s were derived via gene duplication, but were not expressed and pseudogenized in normal cultivated rice during sequence evolution.

## 1. Introduction

Plants have developed various organs, forms of stress tolerance, and environmental adaptability by increasing their genome sizes during evolution [1]. Whole-genome duplication or segmental, tandem, and transposon-mediated gene duplications contribute to increasing the functional diversity of genes [1,2,3,4]. While gene duplication will lead to two identical copies of a gene, the fates of the sister genes differ with subsequent sequence evolution [5,6]. In pseudogenization, one of the sister genes can become a pseudogene by accumulating deleterious mutations during evolution, resulting in gene loss. With gene conservation, both sister genes maintain their function, which can lead to a gene dosage effect. In neofunctionalization, one of the duplicated genes gains a novel function during sequence evolution. In subfunctionalization, the duplicated genes complement each other and maintain their function. Subfunctionalization and neofunctionalization work in a coordinated fashion to generate two novel copies that differ from each other [6,7]. In rice, more than 3,600 pairs of tandem and segmental genes have been identified, excluding transposon genes, such as cytochrome p450, peroxidase, and several transcription factors [8]. These duplicated genes regulate many biological events to adapt to environmental stimuli [9]. Among these duplicated genes, rice genes containing the APETALA2/ethylene-responsive factor (AP2/ERF) domain have increased in number via segmental duplication [8]. The rice AP2/ERF superfamily has more than 130 members scattered throughout the rice genome (Figure S1) [10]. There are signs that the number of genes in several regions has increased via tandem duplication (Figure S1). Genes containing the AP2/ERF domain encode transcription factors and AP2/ERF is divided into four subfamilies based on the domain structure: AP2, AP2/ERF, RAV, and Soloist [10,11]. Their functions are very diverse and include environmental stress, growth, development, senescence, fruit ripening, and defense responses [12]. Rice that possesses short-term flooding tolerance has three *Sub1* genes containing the AP2/ERF domain in tandem on Chromosome 9 (Figure S1) [13]. Of these, *Sub1A* contributes to submergence tolerance, and non-tolerant rice lacks this gene, although it has two similar genes: *Sub1B* and *Sub1C* [13,14]. This suggests that the other two genes were pseudogenized and underwent gene loss or neofunctionalization rather than contributing to submergence tolerance in non-tolerant rice [14]. Another submergence-tolerant rice, deepwater rice, can elongate its internodes in the vegetative stage to avoid anoxia during long-term or stagnant flooding. Elongated internodes keep the top leaves above the water surface, facilitating respiration. In comparison, normal cultivated rice drowns and dies because it cannot elongate its internodes in response to submergence in the vegetative stage despite the various water-tolerant mechanisms in its leaves and roots [15,16,17,18]. We previously identified two factors that enhance the internode elongation of deepwater rice: *SNORKEL1* (*SK1*) and *SNORKEL2* (*SK2*) [19]. *SK* genes possess an AP2/ERF domain involved in ethylene signal transduction, and its expression is induced by ethylene treatment or submergence in deepwater rice [19]. On the other hand, it has been reported that normal cultivated rice lacks the genomic region where the *SK* genes are located, and thus does not elongate under submergence [19]. However, in this study, we have examined the flanking sequences of the SK region, which is missing in normal cultivated rice, and found that normal cultivated rice also possesses *SK-LIKE* genes in the sequences. We then evaluated the gene expression and functions of the *SK-LIKE* genes.

## 2. Results

### 2.1. Sequence Comparison in the SK1/SK2 Region

**Figure 1:**
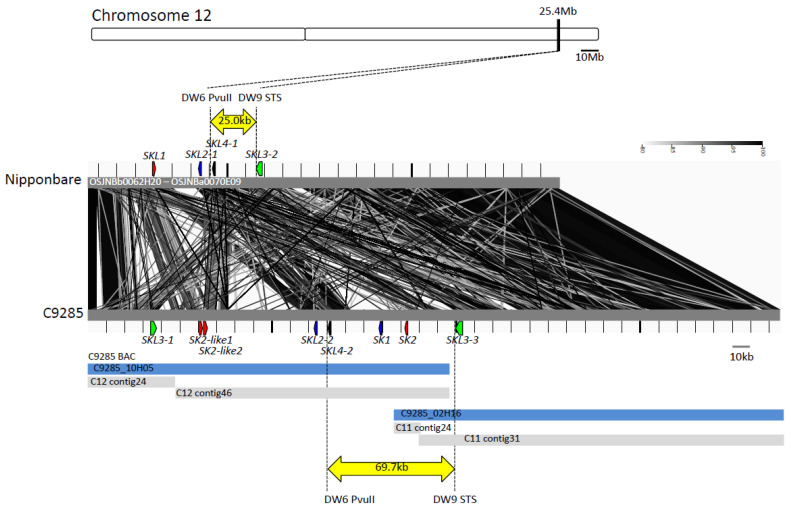
Sequences of the *SNORKEL* genes regions. Yellow arrows indicate candidate regions of *SNORKEL* genes from positional cloning (Hattori et al., 2009). The blue bars represent the BAC sequence of C9285 and the gray bars represent contig sequences. The Nipponbare sequence is derived from IRGSP-1.0. The green, red, blue, and black arrows indicate *SNORKEL* and *SNORKEL-LIKE* genes.

Previously, we identified *SK1* and *SK2*, which encode AP2/ERF domains containing transcription factors as the causal genes of a quantitative trait locus (QTL) on Chromosome 12 that regulates total internode length (TIL) in deepwater rice [*O*. *sativa* admixture C9285 (Dowai38/9)] [19,20,21]. These genes are absent in normal cultivars [*O*. *sativa* ssp. *japonica* Taichung 65 (T65) and Nipponbare (*O*. *sativa* ssp. *japonica*)] because of the deletion of 44.7kb around the *SK* genes (Figure 1) [19,22]. Nevertheless, three AP2/ERF domains containing genes were predicted in the normal cultivated rice Nipponbare in the terminal region on Chromosome 12, which is homologous to the *SK* genes region of deepwater rice (Supplementary Figure S1). To elucidate the chromosome structure of the region, we compared the sequences around the *SK* genes (25.4 M region on Chromosome 12) in the normal cultivated rice—Nipponbare, and the deepwater rice—C9285. First, we selected two bacterial artificial chromosome (BAC) clones that contained the *SK1* and *SK2* genes from Chromosome 12 of C9285 (C9285_10H05 and C9285_02H16). Each BAC clone sequence was assembled into two contigs (C12 contig 24 and C12 contig 46 of C9285_10H05, C11 contig 24 and C11 contig 31 of C9285_02H16). The predicted sequence lengths were 196,017 bp (C9285_10H05) and 208,199 bp (C9285_02H16) (Figure 1). These BAC clone sequences overlapped by approximately 28.4 kb and sufficiently cover the *SK* region on Chromosome 12 in C9285. Next, we obtained Nipponbare BAC clone sequences (OSJNBb0062H20 and OSJNBa0070E09) that corresponded to the *SK* gene region of C9285. Then, we compared the gene structure of the region. Although the upstream and downstream sequences of the *SK* region had highly conserved structures in C9285 and Nipponbare, the region neighboring the *SK* genes contained much variation, such as insertions, deletions, inversions, substitutions, and translocations (Figure 1), implying that genomic reorganization specifically occurred in the *SK* region. Furthermore, the region was highly conserved in another normal cultivated rice, T65, and in deepwater rice (Bhadua: *O*. *sativa* admixture, Appendix A) (Figure S2). One accession of the wild rice *O*. *rufipogon* (W0120) can elongate in response to submergence, and it also has *SK* genes on Chromosome 12 [19,20]. However, it has many polymorphisms compared with the C9285 sequence (Figure S3).

### 2.2. Gene Prediction in the Region around the SK Genes and Phylogenetic Analysis

**Figure 2:**
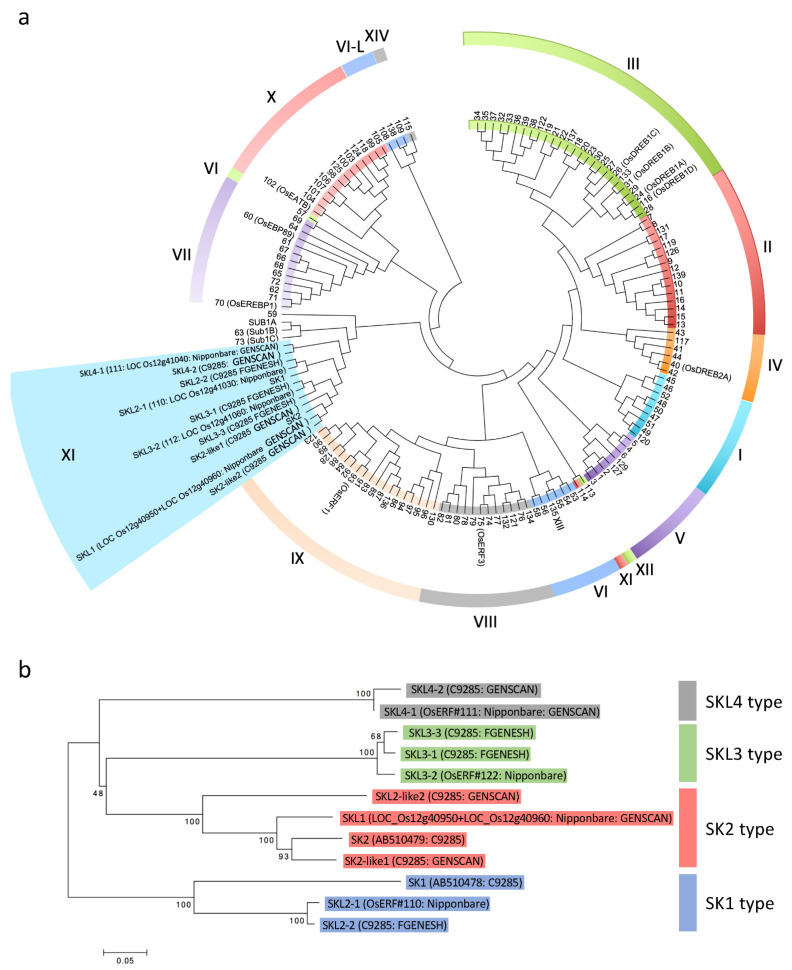
Phylogenetic tree of OsERFs and SNORKEL proteins. (**a**) Phylogenetic tree of OsERFs and SNORKEL proteins based on a comparison of the AP2/ERF domain of OsERFs. The amino acid sequences were aligned using ClustalW and the phylogenetic tree was constructed using the neighbor-joining method. The names of the *ERF* genes and the numbers of subgroups are based on Nakano et al. [10]. (**b**) Phylogenetic tree of SNORKEL and SNORKEL-LIKE proteins, organized by ERF domain sequences.

Despite the absence of *SK* genes in normal cultivated rice due to deletion of the *SK* region, AP2/ERF genes that are similar to *SKs* are located near the end of Chromosome 12 (Figure S1) [10]. To clarify how many AP2/ERF genes are located in the reorganization region, we performed gene prediction using GENSCAN (http://genes.mit.edu/GENSCAN.html, accessed on 20 December 2017) and FGENESH (http://www.softberry.com, accessed on 20 December 2017). This detected six AP2/ERF genes other than *SK1* and *SK2* in this region of the C9285 sequence (Figure 1 and Table 1). In comparison, four AP2/ERF genes were predicted in Nipponbare (Figure 1 and Table 1). None of these genes identified in the reorganization region or other AP2/ERF genes were predicted in the highly conserved regions up- and downstream of the reorganization region. To confirm the relationship between these predicted genes and *SK1*/*SK2*, we performed phylogenetic analysis using the amino acid sequences of the rice AP2/ERF domain (Figure 2a). This showed that all of the predicted genes belonged to the same clade as *SK1*/*SK2*, group XI, and all of the predicted genes contained a nuclear localization signal (Figure 2a and Figure S4) [10]. Therefore, we named these genes *SNORKEL-LIKE* (*SKL*) genes (Table 1). Next, we constructed a phylogenetic tree using the full-length amino acid sequences of *SKs* and *SKLs* to classify these genes in detail. This showed that the *SKs* and *SKLs* could be classified into four subgroups, which we named types SK1 to SKL4 (Figure 2b).

The SK1 subgroup contains three genes: *SKL2-1*^Nipponbare^, *SK1*, and *SKL2-2*^C9285^ (Figure 2b and Table 1). The amino acid sequence homologies of SKL2-1^Nipponbare^ /SK1^C9285^ and SKL2-2^C9285^/SK1^C9285^ were 47.1% and 47.4%, respectively, while SKL2-1^Nipponbare^ /SKL2-2^C9285^ had 97.9% homology (Figure S4a). In addition, the sequences neighboring *SKL2-1*^Nipponbare^ and *SKL2-2*^C9285^ showed high homology, while the sequence neighboring *SK1* did not (Figure S5a). In contrast, the homologies of the AP2/ERF domains of SK1^C9285^/SKL2-1^Nipponbare^, SK1^C9285^/SKL2-2^C9285^, and SKL2-1^Nipponbare^/SKL2-2^C9285^ were 63.3%, 63.3%, and 93.4%, respectively (Figure S4a).

The SK2 subgroup has four homologous genes (Figure 2b). Since GENSCAN predicted that two genes in the Nipponbare sequence database (LOC_Os12g40950 and LOC_Os12g40960) are actually one gene, we named this *SKL1*^Nipponbare^ (Figure S7a and Table 1). GENSCAN predicted that *SKL1*^Nipponbare^ included these two genes and an intermediate sequence as a single gene containing the entire AP2/ERF domain (Figure S7a and Table 1). The *SKL1*^Nipponbare^ intron contains a repeated sequence interspersed in the rice genome. The entire amino acid sequence of SKL1^Nipponbare^ showed 69% homology with the SK2 sequence. In particular, the AP2/ERF domains of SKL1^Nipponbare^ and SK2 shared the same sequence, except for one amino acid substitution (Figure S4b). These results suggest that SKL1^Nipponbare^ functions like SK2 in promoting internode elongation. Further, it was predicted that two *SK2-like* genes were located in tandem in the C9285 genome (Figures S1 and S5b). The entire SK2-like1^C9285^ amino acid sequence showed 72.6% homology with SK2, and the AP2/ERF domain shared 95.0% homology. Although SK2-like2^C9285^ also showed high homology with SK2 in the AP2/ERF domain (95.0%), its entire amino acid sequence showed low homology (53.1%; Figure S4b). The N-end region of *SK2-like2^C9285^* contained repeated sequences that are scattered throughout the genome. Therefore, the N-end amino acid sequence of SK2-like2 differed from the other SK2-type sequences (Figure S4b). No homology was found between the downstream sequences of *SK2* and *SK2-like2^C9285^* (Figure S5b); however, the downstream sequence of *SK2-like2^C9285^* was highly homologous to the neighboring sequence of *SKL4-1*^Nipponbare^, and a 31 kb insertion containing *SKL2-1*^Nipponbare^ and *SKL4-1*^Nipponbare^ was detected in Nipponbare (Figure S5c). The tandem arrangement of *SK2-like1* and *SK2-like2* was also found in the wild rice *O*. *rufipogon* (W0120) (Figure S3), suggesting that the 31 kb insertion occurred in Nipponbare.

The SKL3 subgroup contains three genes (Figure 2b). *SKL3-1* and *SKL3-3* were predicted in the C9285 genome, and *SKL3-2* in the Nipponbare genome. The total amino acid homologies of SKL3-1*^C9285^*/SKL3-2^Nipponbare^, SKL3-1*^C9285^*/SKL3-3*^C9285^*, and *SKL3-2^Nipponbare^* /SKL3-3*^C9285^* were 92.9%, 95.3%, and 92.3%, respectively (Figure S4c). The sequences of the AP2/ERF domain matched completely (Figure S4c). The sequences flanking the *SKL3*s were highly conserved, although some insertions and deletions were detected (Figure S5d). To distinguish *SKL3-1* and *SKL3-3*, we designed genotyping markers (SKL3 check F and SKL3 check R) (Figure S5d). For genotyping, we used two types of normal cultivated rice (Nipponbare and T65) and two of deepwater rice (C9285 and Bhadua). We also used two BAC clones of C9285, which contain only a single *SKL3* in each sequence (C9285_10H05 and C9285_02H16; Figure 1). Genotyping detected only one band by PCR using genomic DNA of normal cultivated rice (Nipponbare and T65), whereas two bands were detected in deepwater rice (C9285 and Bhadua) (Figure S5e). In addition, a single band corresponding to *SKL3-1* was amplified in the BAC clone C9285_10H05, while another single band corresponding to *SKL3-3* was detected in the BAC clone C9285_02H16 (Figure 1 and Figure S5e). These results suggest that two *SKL3* genes (*SKL3-1* and *SKL3-3*) are present in duplicate in the deepwater rice genome.

The SKL4 subgroup contained two genes (*SKL4-1^Nipponbare^* and *SKL4-2^C9285^*) (Figure 1 and Figure 2b). It was predicted that *SKL4-2^C9285^* comprised two exons (Figure S5f). The amino acid sequences of SKL4-1^Nipponbare^ and SKL4-2^C9285^ were highly conserved between normal cultivated rice and deepwater rice, except for Exon 1 of *SKL4-2* (Figures S4d and S5f). There was a 10.4-kb insertion or deletion between the upstream region of *SKL4-1^Nipponbare^* or the intron of *SKL4-2^C9285^*, resulting in a completely different sequence for Exon 1 of *SKL4-2^C9285^* compared to *SKL4-1^Nipponbare^* (Figures S4d and S5f). The inserted *SKL4-1^Nipponbare^* sequence showed high homology with the upstream sequence of *SKL3-3^C9285^* in deepwater rice (Figure S5f). A BLAST search based on the *SKL4-1^Nipponbare^* insertion sequence showed high homology with the transposon protein CACTA, En/Spm sub-class. Although GENSCAN predicted that *SKL4-1^Nipponbare^* was a 594 bp gene containing an AP2/ERF domain, the Nipponbare database predicted a 7020 bp gene containing a transposon sequence and AP2/ERF domain in this region (LOC_Os12g41040) (Table 1 and Figure S5f). These results suggest that *SKL4-1^Nipponbare^* was disrupted by insertion of a transposon element.

### 2.3. SK and SKL Gene Expression

**Figure 3:**
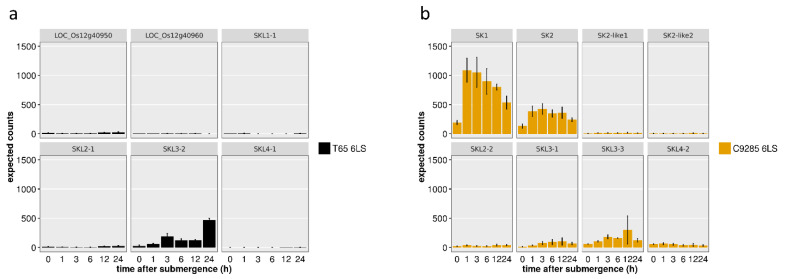
Expression of *SNORKEL* and *SNORKEL-LIKE* genes. Expression of each gene in (**a**) normal cultivated rice (T65) and (**b**) deepwater rice (C9285) during submergence. Gene expression levels were extracted from the data of a previous RNA-Seq analysis [23]. Rice seedlings were completely submerged for 1, 3, 6, 12, and 24 h and data from plants at the six-leaf stage were employed for this analysis. Data are mean ± S.D. (n = 3 plants).

To confirm the expression levels of *SK*s and *SKL*s, we used open RNA-Seq data for normal cultivated (T65) and deepwater (C9285) rice [23]. This dataset contains temporal expression data under submergence, and the *SKL* expression levels were quantified by referring to the genome sequences of the predicted genes. The gene expressions of *SK1* and *SK2* increased rapidly within an hour of being submerged, consistent with reports that *SK1* and *SK2* expression is induced by deepwater conditions (Figure 3 and Figure S6) [19]. In T65 and C9285, the expression of *SKL3*-2 in T65 and *SKL3-3* in C9285 were also induced by submergence, and the expression patterns were similar in both rice (Figure 3 and Figure S6). The expression of other genes was either extremely low or could not be observed. Neither the full-length *SKL1* in T65 nor the two genes constituting *SKL1* (LOC_Os12g40950 and LOC_Os12g40960) showed altered expression under deepwater conditions (Figure 3a).

### 2.4. Effects of SKs and SKL1 on Internode Elongation

**Figure 4:**
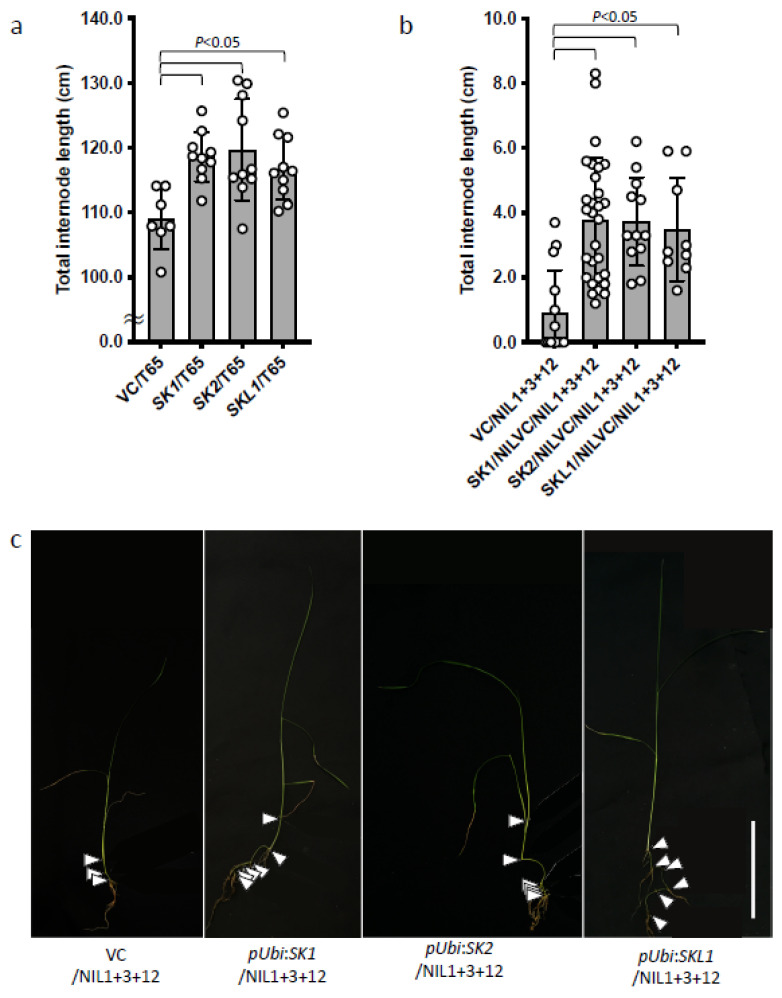
The function of SKL1. (**a**) Total internode length of plants that overexpress *SK1*, *SK2*, or *SKL1* in T65 after maturation. (**b**) Total internode length of plants that overexpress *SK1*, *SK2*, or *SKL1* in NIL1 + 3 + 12 during the vegetative phase. Dots indicate the total internode lengths of individual plants. Data are mean ± S.D (n ≥ 5 in (**a**) and n ≥ 9 in (**b**). Two-tailed *t*-test compared with the vector control (VC) in T65 (**a**) or in NIL1 + 3 + 12 (**b**). (**c**) T_0_ plants overexpressing *SK1*, *SK2*, or *SKL1* in NIL1 + 3 + 12. Arrowheads indicate nodes linked by elongated internodes. Scale bars: 10 cm.

It has been reported that plants overexpressing *SK1* and *SK2* undergo internode elongation under normal growth conditions, and that *SK2* has a greater effect on internode elongation than *SK1* [19]. *SKL1* expression of T65 was not upregulated under deepwater conditions, but it had the highest homology to *SK2* (Figure 3 and Figure S4). Therefore, we generated transgenic plants overexpressing *SKL1* to test whether *SKL1* functions in internode elongation. Since *SKL1* is not expressed in T65 under deepwater conditions, we amplified each exon by PCR and linked them to obtain the full-length sequence of *SKL1* (Figure S7). As transgenic backgrounds, we used normal cultivated rice, T65, and a nearly isogenic line (NIL) 1 + 3 + 12 that contained three major QTLs related to internode elongation under deepwater conditions [19]. The plants overexpressing *SK1*, *SK2*, or *SKL1* in T65 had longer internodes than the vector control in the reproductive stage (Figure 4a). The vector control plants in the NIL1 + 3 + 12 background showed little internode elongation in the early vegetative phase, whereas lines overexpressing *SK1*, *SK2*, or *SKL1* showed significant internode elongation during this phase (Figure 4b,c). These results suggest that SKL1 protein possesses the ability to elongate internodes as SK1 and SK2.

### 2.5. Validation of SKL1 Transcriptional Activity

**Figure 5:**
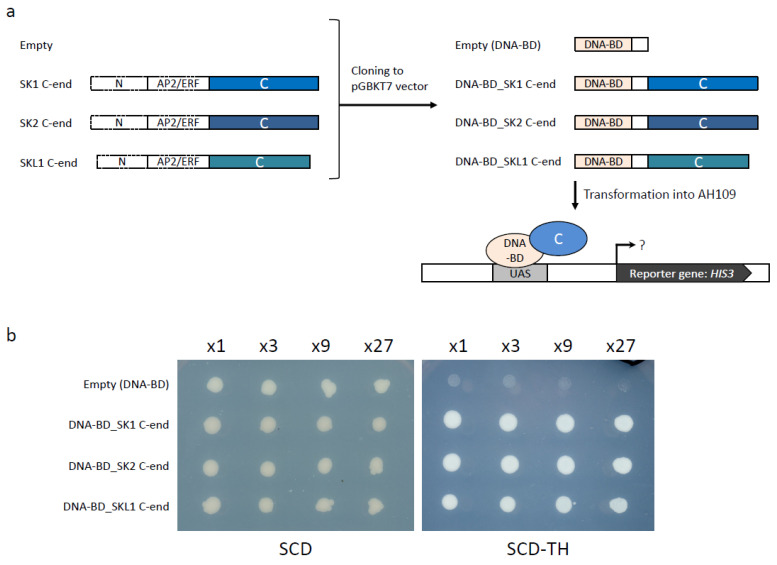
Transcription activity of *SK1*, *SK2*, and *SKL1*. (**a**) Schematic representation of the yeast one-hybrid assay. Left: PCR amplification of the C-end sequences of SK1, SK2, and SKL1. Right: each C-end sequence is ligated to the pGBKT7 vector to create a fusion protein with the GAL4 DNA-binding domain. Bottom: a schematic of *HIS3* reporter gene transactivation by DNA-BD_C-end. (**b**) Transactivation activity assay in yeast. The experiment was repeated three times with similar results.

It was reported that the C-end regions of SK1 and SK2 have transcriptional activity [19]. Since the *SKL1* overexpression line induced internode elongation (Figure 4), we performed a yeast one-hybrid assay to verify the transcriptional activity of *SKL1*. The C-end sequences of SK1, SK2, and SKL1 were ligated to pGBKT7 to fuse the GAL4 DNA-binding domain (Figure 5a). The C-end regions of SK1, SK2, and SKL1 induced expression of the *HIS3* reporter gene (Figure 5b), suggesting that there is transcriptional activity in the C-terminal region of SKL1, as well as in the C-terminal regions of SK1 and SK2. These results suggest that T65 is deficient in the gene expression of *SKL1*, but the protein itself of SKL1 has the same ability to promote transcription as SK1 and SK2.

## 3. Discussion

The rice genome has more than 130 transcription factors containing the AP2/ERF domain [10]. The AP2/ERF family is involved in many biological phenomena, such as flower development, senescence, fruit ripening, and biotic and abiotic stress responses [12]. We previously reported that *SK1* and *SK2*, which contain AP2/ERF domains, regulate internode elongation in deepwater rice in response to submergence [19]. However, these genes do not exist in normal cultivated rice as a result of a large chromosome segment deletion [19]. To clarify the details of the sequence structure of the *SK1* and *SK2* region in normal cultivated and deepwater rice, we conducted a comparative sequence analysis and gene prediction of the *SK*s region. We identified four novel putative AP2/ERF domain-containing genes in normal cultivated rice and six in deepwater rice (Figure 1, Table 1). Phylogenetic analysis using the amino acid sequences of the AP2/ERF domains revealed that the SKs and predicted AP2/ERF genes belong to the same subfamily (group XI), although over 130 AP2/ERF genes have been reported (Figure 2 and Figure S1) [10]. Furthermore, the genes of group XI including *SK*s clustered only on Chromosome 12 (Figure 2), suggesting that the predicted genes had the same origin and differentiated via multiple gene duplications in specific regions of Chromosome 12.

Gene duplication leads first to the acquisition of a dual function. In addition to cases in which genes with functional duplication are retained (conserved) in subsequent processes, various other types of functional differentiation can occur [24]. When unfavorable mutations accumulate in one of the genes generated by gene duplication, the gene becomes non-functional and is a pseudogene. However, sequence evolution may cause a new function to arise in one of the sister genes (neofunctionalization) or differentiation into two genes with duplicated function (subfunctionalization). These phenomena have also been reported in enzymes. Cytochrome p450 genes have established various metabolic pathways via sequence duplication and evolution in plants [25]. Another example of functional differentiation after gene duplication has been reported for the *Sub1* genes, which belong to the same ERF family as SKs. The *Sub1* region of submergence-tolerant lines such as FR13A consists of three tandem duplication genes: *SUB1A*, *SUB1B*, and *SUB1C* [13,14]. Even submergence-intolerant lines, such as Nipponbare, possess two tandemly duplicated genes: *SUB1B* and *SUB1C*. However, among the three genes, only *SUB1A* functions as a submergence-tolerant factor. This suggests that functional differentiation occurred at the gene duplication site (neofunctionalization) or that the function was lost through amino acid mutation in sequence evolution (non-functionalization) [14]. Likewise, there are multiple genes containing the AP2/ERF domain in the *SK* gene regions of both normal cultivated and deepwater rice, but the only genes whose expression increased under submergence were *SK1*, *SK2* in deepwater rice and *SKL3*s in both varieties (Figure 3 and Figure S6). However, normal cultivated rice does not elongate its internodes under water despite expressing *SKL3* (Figure 3a). These results imply that the *SK-LIKE* genes other than *SK1* and *SK2* were non-functionalized and pseudogenized, or that the genes are involved in physiological phenomena other than submergence via neofunctionalization.

Of the predicted genes, *SKL1* was predicted to be two separate genes (LOC_Os12g40950 and LOC_Os12g40960) in the Nipponbare genome (Figure S7a and Table 1). However, GENSCAN detected one gene that spanned both of these genes and contained the entire AP2/ERF domain (Figure S6 and Table 1). The upstream sequences of *SKL1* and *SK2* were very similar, and the SKL1 amino acid sequence showed the highest homology to SK2 (Figure 2, Figures S4 and S5). T65 transgenic plants overexpressing *SK1*, *SK2*, and SKL1 had longer internodes than VC during the reproductive phase (Figure 4c). In addition, overexpression of SKL1 in NIL1 + 3 + 12, which contains three major QTLs associated with internode elongation, induced internode elongation in the vegetative phase as well as in lines overexpressing *SK1* and *SK2* (Figure 4b,c). Although T65 transgenic plants overexpressing *SK1*, *SK2,* and *SKL1* showed internode elongation in the reproductive stage, this ability seemed to be lower than in NIL1 + 3 + 12 transgenic plants. NIL1 + 3 + 12 has the Chromosome 1 segment of deepwater rice containing *GA20ox2* and submergence induced the expression of the *GA20ox2* allele in C9285 [22]. In addition, *GA20ox2* (*indica* type allele) in C9285 has greater enzymatic activity than that of the *japonica* type due to two amino acid substitutions [22,26]. C9285 had the *indica* type *GA20ox2*, while T65 had the *japonica* type [22]. Additionally, NIL1 + 3 + 12 has the causal gene of the QTL associated with the initiation of internode elongation on Chromosomes 3 and 12 of deepwater rice encode *ACCELERATOR OF INTERNODE ELONGATION 1* (*ACE1*) and *DECELERATOR OF INTERNODE ELONGATION 1* (*DEC1*), respectively [27]. The deepwater rice type ACE1 promotes the initiation of internode elongation in response to gibberellins. By contrast, DEC1, a repressor of internode elongation, is downregulated in response to gibberellins in deepwater rice. In normal cultivated rice, gibberellin levels increase during the reproductive phase, and expression of the *ACE1* homolog *ACE1-LIKE1* increases, while *DEC1* expression is repressed, leading to the initiation of internode elongation. These results suggest that *SKL1*, like *SK1* and *SK2*, promotes the elongation of initiated internodes rather than hastening the onset of internode elongation. Promoting *ACE1* expression and repressing *DEC1* expression with gibberellins initiates internode elongation, and SKs enhance internode length. SK1 and SK2 have transcription activity in the C-terminal region [19]. SKL1 also possesses transcriptional activity in the C-terminal region (Figure 5). These results suggest that *SKL1* and *SK2* have the same origin, although *SKL1* might have been pseudogenized during sequence evolution. Recently, it was reported that *Arabidopsis* ERF11 promotes internode elongation by indirectly activating gibberellin biosynthesis [28]. Furthermore, AtERF11 has been shown to directly bind to the DELLA protein, which is a gibberellin signaling suppressor, thereby inhibiting DELLA function and promoting stem elongation [28]. Since SKs also belong to the AP2/ERF family, they may induce internode elongation by suppressing rice DELLA protein SLR1 function via interaction with SKs-SLR1 in addition to the transcriptional activity of SK1, SK2, and SKL1^Nipponbare^.

Generally, taller rice is more susceptible to being blown over by wind or lodged by rain than shorter rice, resulting in yield losses. Therefore, ancient farmers might have selected shorter, non-lodging rice. Indeed, it is reported that shorter rice related to gibberellin biosynthesis (GA20ox-2 (*sd1*)) was selected artificially during the domestication process [22,26,29,30]. Regarding *SK* genes, we revealed that *SK-LIKE* genes also exist tandemly in one accession of the wild rice *O*. *rufipogon* (W0120), which initiates internode elongation in response to submergence (Figure S3) [19]. However, the elongation ability via *SK* genes might have been selected from wild rice such as *O*. *rufipogon* (W0120) for domestication in areas that flood. Further research using numerous *O*. *rufipogon* accessions will reveal the relationship between SKs and the domestication process.

## 4. Materials and Methods

### 4.1. Construction of the BAC Library and Sequencing of BAC Clones

Rice DNA was isolated from young leaves of normal paddy rice—T65; deepwater rice—C9285 and Bhadua; and wild rice—W0120 (*O. rufipogon*), using a method described previously [31]. Positive BAC clones completely covering the gene region were subjected to capillary sequencing (ABI3730; Applied Biosystems, Foster, CA, USA) using a shotgun strategy as described previously [32].

### 4.2. Gene Prediction and Sequence Comparison

Genes within the genome sequences of T65, C9285, Bhadua, and W0120 were predicted using the GENSCAN (http://genes.mit.edu/GENSCAN.html, accessed on 20 December 2017) and FGENESH (http://www.softberry.com, accessed on 20 December 2017) tools. Among the predicted genes, those containing AP2/ERF domains were considered to be *SK-LIKE* gene candidates. GenomeMatcher was used to compare the genome sequences [33]. Alignments of the gene coding sequences (CDSs) and amino acid sequences were performed using Genetyx software (ver. 14.0.0; GENETYX Corp., Tokyo, Japan).

### 4.3. Gene Expression Analysis

We estimated the expression levels of *SK*s and *SK-LIKE* genes in T65 and C9285 using previously published RNA-sequencing data comparing non-deepwater rice (T65) with deepwater rice (C9285) [23]. Rice seedlings were completely submerged for 1, 3, 6, 12, and 24 h and data from plants at the six-leaf stage were employed for expression analysis.

### 4.4. Production of Transgenic Plants

To overexpress *SK1*, *SK2*, and *SKL1*, the CDS fragments of each gene were amplified and fused to pCAMBIA1380 containing the maize (*Zea mays*) *UBIQUITIN1* promoter. The amplification of the *SKL1* CDS is depicted in Supplementary Figure S7. The primers used are listed in Supplementary Table S1. The resulting constructs were introduced into a T65 or NIL1 + 3 + 12 containing three major QTLs related to internode elongation under deepwater conditions [19] by *Agrobacterium tumefaciens* (EHA105)-mediated transformation [34].

### 4.5. Plant Growth Conditions

The transgenic plants (T_0_) were transplanted in perforated plastic pots (9 × 9 × 12 cm) filled with soil (N, P, and K at 0.25, 0.3, and 0.25 g/kg, respectively; Aichi Medel Corp., Japan) and grown in a greenhouse in natural light conditions at Nagoya University, Japan. The water level in the pots was maintained at ~5 cm above the soil surface (shallow-water conditions).

### 4.6. Transcriptional Activity Assay

A yeast one-hybrid system was employed to investigate transcriptional activation driven by SK using the reporter gene (*HIS3*) with the C-termini of *SK1, SK2*, and *SKL1.* These fragments were fused to the GAL4-DNA binding domain in pGBKT7 (Clontech-Takara Bio, Tokyo, Japan). The resulting plasmids were transformed into the yeast strain AH109 (Takara Bio, Tokyo, Japan). The yeast liquid cultures were diluted to an absorbance at 600 nm of 0.6, and 2 μL of each dilution were inoculated onto tryptophan- and histidine-negative synthetic dropout medium.

### 4.7. Statistics and Reproducibility

Two-tailed *t*-tests were used to evaluate significance, and were performed using Prism 7 software. The calculated *p* values are shown in each graph above the line that connects the two datasets. Measurements were performed by randomly selecting plants grown under exactly the same conditions. All samples were allocated randomly to experimental groups.

## 5. Conclusions

*SNORKEL1* and *SNORKEL2* are exclusively present in the genomes of deepwater rice, where they promote internode elongation. In this study, several genes similar to the *SNORKEL* genes were detected in normal cultivated rice. However, these genes were not expressed under submergence conditions. When SKL1, which has the highest sequence similarity to *SNORKEL2*, was artificially expressed in the normal cultivated rice strains, internode elongation was promoted during the reproductive phase in strain T65 and during the vegetative phase in strain NIL1 + 3 + 12. These results suggest that multiple *SK-LIKE* genes, including the *SNORKEL*s, were generated by gene duplication in the region, resulting in non- or neofunctionalization.

## Figures and Tables

**Table 1:** List of *SNORKEL* and *SNORKEL-LIKE* genes.

Gene Name	Gene Name	Gene Name	Gene Name	Gene Name	Gene Name	Gene Name
*SK1*	C9285	-	771	-	-	AB510478
*SK2*	C9285	-	777	-	-	AB510479
*SKL1*	Nipponbare	LOC_Os12g40950	417	666	-	GENSCAN
	Nipponbare	LOC_Os12g40960	198		-	
*SK2-like1*	C9285	-	-	726	-	GENSCAN
*SK2-like2*	C9285	-	-	831	-	GENSCAN
*SKL2-1*	Nibbonbare	LOC_Os12g41030	423	-	OsERF#110	-
*SKL2-2*	C9285	-	-	423	-	FGENESH
*SKL3-1*	C9285	-	-	969	-	FGENESH
*SKL3-2*	Nipponbare	LOC_Os12g41060	972	-	OsERF#112	AK242027
*SKL3-3*	C9285	-	-	972	-	FGENESH
*SKL4-1*	Nipponbare	LOC_Os12g41040 *	7020	594	OsERF#111	GENSCAN
*SKL4-2*	C9285	-	-	762	-	GENSCAN

Gene prediction was based on GENSCAN and FGENESH. Length is based on MUS; predicted length means the predicted size of genes. * LOC_Os12g41040 contains a transposon-like sequence in the MUS database, but here the sequence predicted by GENSCAN was used as SKL4-1. ERF numbers are based on Nakano et al. (2006).

## Data Availability

The sequence data presented in this study are available in DNA Database of Japan (DDBJ). The RNA-seq data used in this study can be found in the DDBJ under bioproject PRJDB5294 (cv C9285 RNA-Seq reads).

## Supplementary Materials

The following supporting information can be downloaded at: https://www.mdpi.com/article/10.3390/plants11030376/s1. Supplementary Figure S1. Location of ERF family genes on Nipponbare chromosomes. Colored boxes indicate tandem duplicated ERF genes. The gene locus IDs follow [10]. The colored squares next to the gene IDs represents ERFs that exist as a cluster, respectively. The gene locations were determined using the chromosome map tool (http://viewer.shigen.info/oryzavw/maptool/MapTool.do, accessed on 20 December 2017). Among the genes listed in Nakano et al. (2006), LOC_Os06g09690 and LOC_Os06g09730 could not be detected with this tool. Supplementary Figure S2. Comparison of multiple sequences of SNORKEL gene regions between normal cultivated rice (Taichung 65 and Nipponbare) and deep water rice (C9285 and Bhadua). Supplementary Figure S3. Sequence comparison between deep water rice (C9285) and wild rice (*O. rufipogon*, W0120). Supplementary Figure S4. Comparison of protein sequences. Protein sequences of the (a) SK1, (b) SK2, (c) SKL3, and (d) SK4 types. The amino acid sequences were aligned using ClustalW. The red and blue dashed lines represent the AP2/ERF domain and nuclear localization signal, respectively. Supplementary Figure S5. Comparison of genomic sequence. Sequence comparison of the genomic regions of the (a) SK1 type, (b) SK2 type, (c) Nipponbare and SK2-like genes of C9285, (d) SKL3 type, (e) SKL3 alleles, and (f) SKL4 type. The gray region in each gene model indicates an intron. Supplementary Figure S6. Representative raw RNA-Seq based on transcripts of deep water rice submerged for 24 hours. Yellow arrows indicate exons. Supplementary Figure S7. Construction of the plasmid for SKL1 overexpression. (a) Gene structure of SKL1, as predicted by GENSCAN. LOC_Os12g40960 harbors a truncated form of the AP2/ERF domain in the N-end region, while LOC_Os12g40950 lacks an AP2/ERF domain in its sequence. (b) Cloning the SKL1 CDS. The red region indicates the AP2/ERF domain. Arrows indicate primers. Exons predicted by GENSCAN were amplified in the first PCR step. The second PCR was performed with the PCR products obtained in the first PCR and Primers 1-F and 2-R. The second PCR product was cloned into the vector for overexpression. Electrophoresis of the (c) first and (d) second PCRs. Supplementary Table S1. Primer list.

## Appendix A

**Accession number:** Bhadua_BAC, LC651146; W0120_BAC_21H19, LC651147; C9285_BAC, LC651148; SK2-like1, LC651149; SK2-like2, LC651150; SKL1, LC651151; SKL2-2, LC651152; SKL3-1, LC651153; SKL3-3, LC651154; SKL4-1, LC651155; SKL4-2, LC651156.

## References

[1] Panchy N., Lehti-Shiu M., Shiu S.H. (2016). Evolution of gene duplication in plants. Plant Physiol..

[2] Rubin G.M., Yandell M.D., Wortman J.R., Gabor Miklos G.L., Nelson C.R., Hariharan I.K., Fortini M.E., Li P.W., Apweiler R., Fleischmann W. (2000). Comparative genomics of the eukaryotes. Science.

[3] Kent W.J., Baertsch R., Hinrichs A., Miller W., Haussler D. (2003). Evolution’s cauldron: Duplication, deletion, and rearrangement in the mouse and human genomes. Proc. Natl. Acad. Sci. USA.

[4] Cannon S.B., Mitra A., Baumgarten A., Young N.D., May G. (2004). The roles of segmental and tandem gene duplication in the evolution of large gene families in Arabidopsis thaliana. BMC Plant Biol..

[5] Lynch M., Conery J.S. (2000). The evolutionary fate and consequences of duplicate genes. Science.

[6] Assis R., Bachtrog D. (2013). Neofunctionalization of young duplicate genes in Drosophila. Proc. Natl. Acad. Sci. USA.

[7] He X., Zhang J. (2005). Rapid subfunctionalization accompanied by prolonged and substantial neofunctionalization in duplicate gene evolution. Genetics.

[8] Jiang S.Y., González J.M., Ramachandran S. (2013). Comparative Genomic and Transcriptomic Analysis of Tandemly and Segmentally Duplicated Genes in Rice. PLoS ONE.

[9] Hanada K., Zou C., Lehti-Shiu M.D., Shinozaki K., Shiu S.H. (2008). Importance of lineage-specific expansion of plant tandem duplicates in the adaptive response to environmental stimuli. Plant Physiol..

[10] Nakano T., Suzuki K., Fujimura T., Shinshi H. (2006). Genome-Wide Analysis of the ERF Gene Family. Plant Physiol..

[11] Licausi F., Giorgi F.M., Zenoni S., Osti F., Pezzotti M., Perata P. (2010). Genomic and transcriptomic analysis of the AP2/ERF superfamily in Vitis vinifera. BMC Genom..

[12] Gu C., Guo Z.H., Hao P.P., Wang G.M., Jin Z.M., Zhang S.L. (2017). Multiple regulatory roles of AP2/ERF transcription factor in angiosperm. Bot. Stud..

[13] Xu K., Xu X., Fukao T., Canlas P., Maghirang-Rodriguez R., Heuer S., Ismail A.M., Bailey-Serres J., Ronald P.C., Mackill D.J. (2006). Sub1A is an ethylene-response-factor-like gene that confers submergence tolerance to rice. Nature.

[14] Fukao T., Bailey-Serres J. (2008). Submergence tolerance conferred by Sub1A is mediated by SLR1 and SLRL1 restriction of gibberellin responses in rice. Proc. Natl. Acad. Sci. USA.

[15] Kurokawa Y., Nagai K., Huan P.D., Shimazaki K., Qu H., Mori Y., Toda Y., Kuroha T., Hayashi N., Aiga S. (2018). Rice leaf hydrophobicity and gas films are conferred by a wax synthesis gene (LGF1) and contribute to flood tolerance. New Phytol..

[16] Yamauchi T., Nakazono M. (2021). Mechanisms of lysigenous aerenchyma formation under abiotic stress. Trends Plant Sci..

[17] Yin Y.G., Mori Y., Suzui N., Kurita K., Yamaguchi M., Miyoshi Y., Nagao Y., Ashikari M., Nagai K., Kawachi N. (2021). Noninvasive imaging of hollow structures and gas movement revealed the gas partial-pressure-gradient-driven long-distance gas movement in the aerenchyma along the leaf blade to submerged organs in rice. New Phytol..

[18] Mori Y., Kurokawa Y., Koike M., Malik A.I., Colmer T.D., Ashikari M., Pedersen O., Nagai K. (2019). Diel O_2_ dynamics in partially and completely submerged deepwater rice: Leaf gas films enhance internodal O2 status, influence gene expression and accelerate stem elongation for ‘snorkelling’ during submergence. Plant Cell Physiol..

[19] Hattori Y., Nagai K., Furukawa S., Song X.-J., Kawano R., Sakakibara H., Wu J., Matsumoto T., Yoshimura A., Kitano H. (2009). The ethylene response factors *SNORKEL1* and *SNORKEL2* allow rice to adapt to deep water. Nature.

[20] Hattori Y., Miura K., Asano K., Yamamoto E., Mori H., Kitano H., Matsuoka M., Ashikari M. (2007). A major QTL confers rapid internode elongation in response to water rise in deepwater rice. Breed. Sci..

[21] Hattori Y., Nagai K., Mori H., Kitano H., Matsuoka M., Ashikari M. (2008). Mapping of three QTLs that regulate internode elongation in deepwater rice. Breed. Sci..

[22] Kuroha T., Nagai K., Gamuyao R., Wang D.R., Furuta T., Nakamori M., Kitaoka T., Adachi K., Minami A., Mori Y. (2018). Ethylene-gibberellin signaling underlies adaptation of rice to periodic flooding. Science.

[23] Minami A., Yano K., Gamuyao R., Nagai K., Kuroha T., Ayano M., Nakamori M., Koike M., Kondo Y., Niimi Y. (2018). Time-course transcriptomics analysis reveals key responses of submerged deepwater rice to flooding. Plant Physiol..

[24] Wagner A. (1998). The fate of duplicated genes: Loss or new function?. BioEssays.

[25] Liu Z., Tavares R., Forsythe E.S., André F., Lugan R., Jonasson G., Boutet-Mercey S., Tohge T., Beilstein M.A., Werck-Reichhart D. (2016). Evolutionary interplay between sister cytochrome P450 genes shapes plasticity in plant metabolism. Nat. Commun..

[26] Asano K., Yamasaki M., Takuno S., Miura K., Katagiri S., Ito T., Doi K., Wu J., Ebana K., Matsumoto T. (2011). Artificial selection for a green revolution gene during japonica rice domestication. Proc. Natl. Acad. Sci. USA.

[27] Nagai K., Mori Y., Ishikawa S., Furuta T., Gamuyao R., Niimi Y., Hobo T., Fukuda M., Kojima M., Takebayashi Y. (2020). Antagonistic regulation of the gibberellic acid response during stem growth in rice. Nature.

[28] Zhou X., Zhang Z.L., Park J., Tyler L., Yusuke J., Qiu K., Nam E.A., Lumba S., Desveaux D., McCourt P. (2016). The ERF11 transcription factor promotes internode elongation by activating gibberellin biosynthesis and signaling. Plant Physiol..

[29] Sasaki A., Ashikari M., Ueguchi-Tanaka M., Itoh H., Nishimura A., Swapan D., Ishiyama K., Saito T., Kobayashi M., Khush G.S. (2002). A mutant gibberellin-synthesis gene in rice: New insight into the rice variant that helped to avert famine over thirty years ago. Nature.

[30] Ashikari M., Sasaki A., Ueguchi-Tanaka M., Itoh H., Nishimura A., Datta S., Ishiyama K., Saito T., Kobayashi M., Khush G.S. (2002). Loss-of-function of a Rice Gibberellin Biosynthetic Gene, GA20 oxidase (GA20ox-2), Led to the Rice ‘Green Revolution’. Breed. Sci..

[31] Zhang H.-B., Zhao X., Ding X., Paterson A.H., Wing R.A. (1995). Preparation of megabase-size DNA from plant nuclei. Plant J..

[32] International Rice Genome Sequencing Project (2005). The map-based sequence of the rice genome. Nature.

[33] Ohtsubo Y., Ikeda-Ohtsubo W., Nagata Y., Tsuda M. (2008). GenomeMatcher: A graphical user interface for DNA sequence comparison. BMC Bioinform..

[34] Hiei Y., Ohta S., Komari T., Kumashiro T. (1994). Efficient transformation of rice (*Oryza sativa* L.) mediated by *Agrobacterium* and sequence analysis of the boundaries of the T-DNA. Plant J..

