# Supplementary material for: SNORKEL Genes Relating to Flood Tolerance Were Pseudogenized in Normal Cultivated Rice
Source: Plants (Basel). 2022 Jan 29;11(3):376. doi: 10.3390/plants11030376 (PMC8840289; doi:10.3390/plants11030376)
Supplement: Supplementary file 1 [file plants-11-00376-s001.zip › Supplementary Figures (Nagai et al).pptx]

## Slide 1
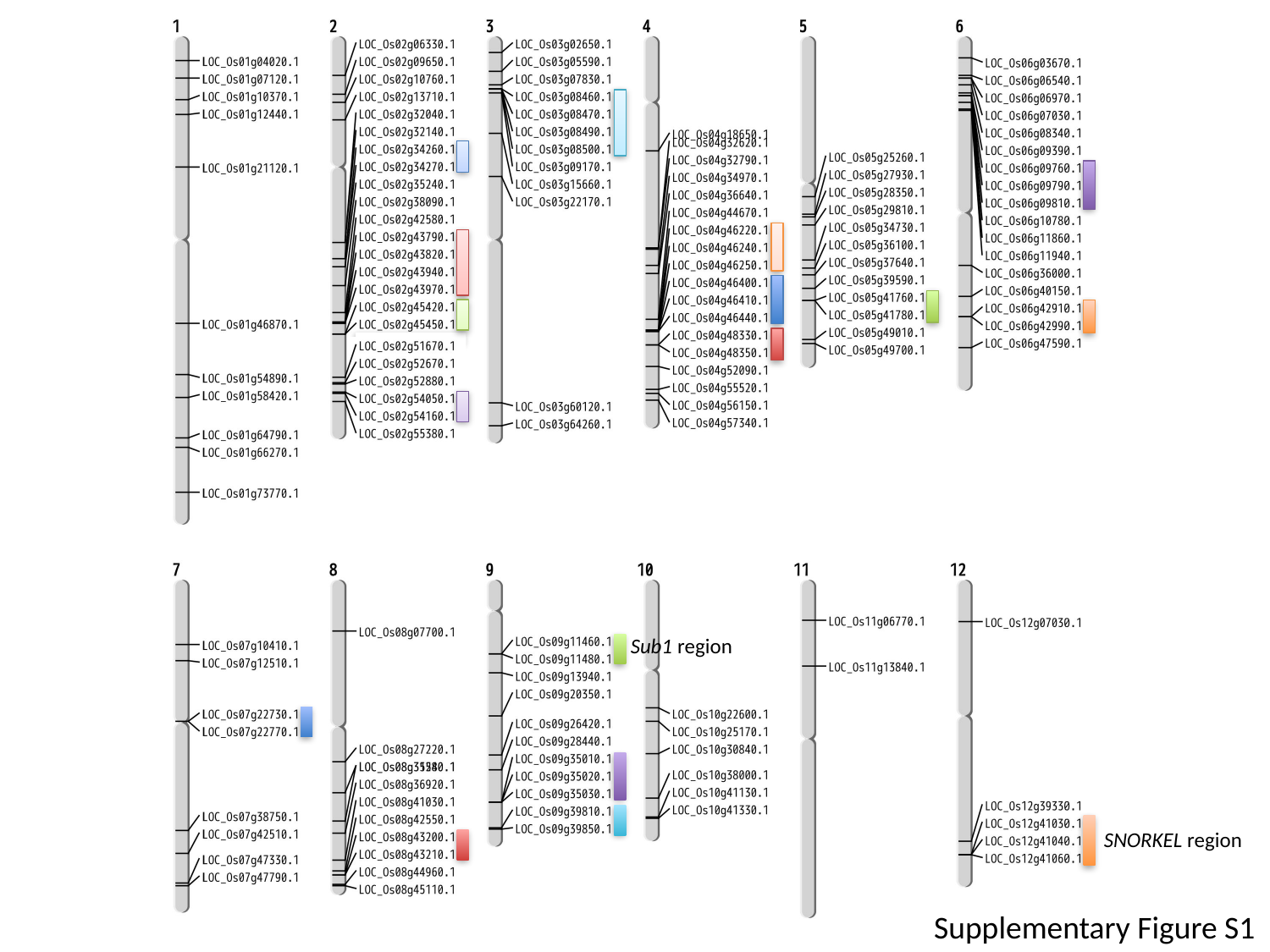

Sub1 region
SNORKEL region
Supplementary Figure S1

## Slide 2
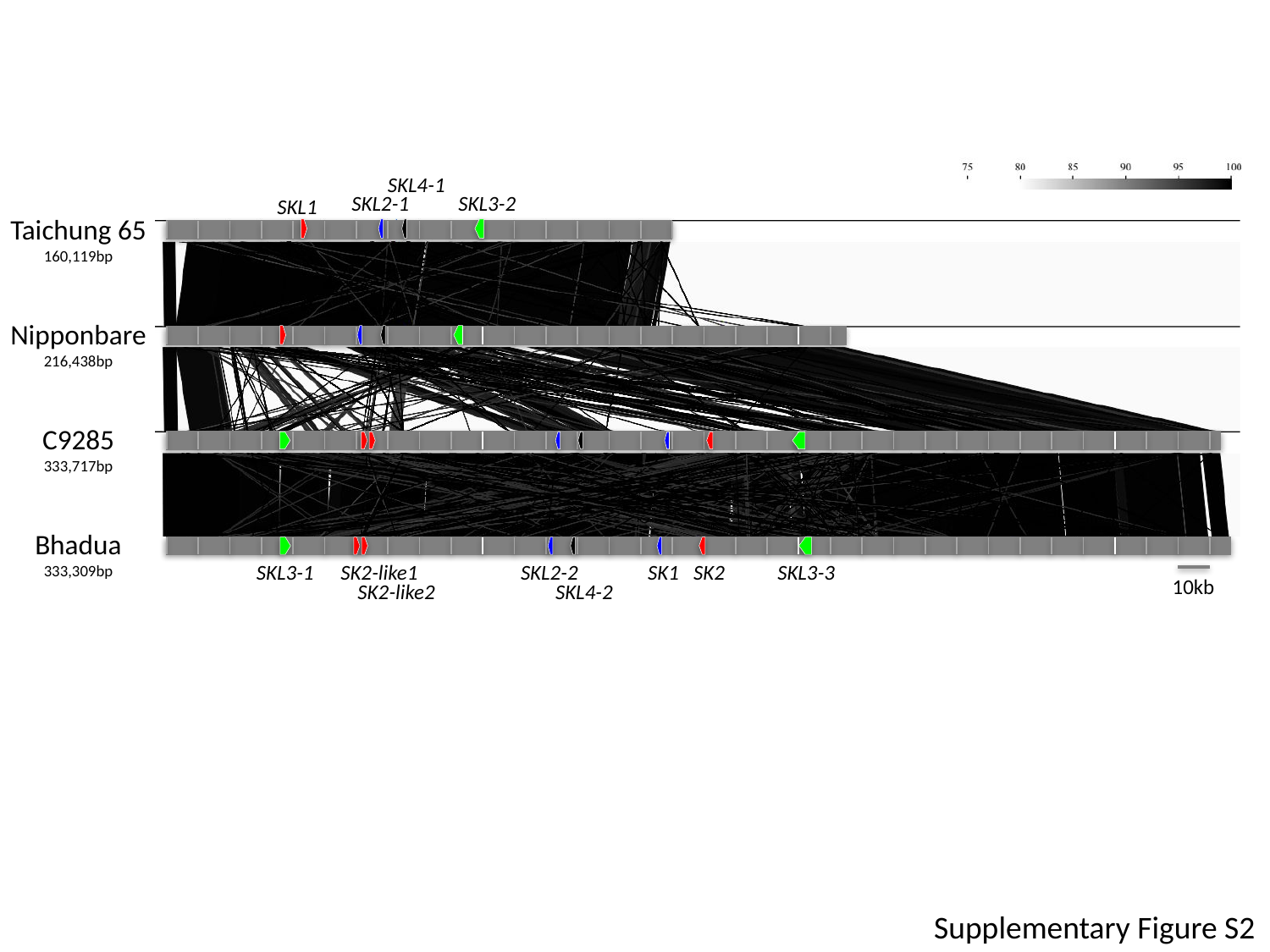

SKL4-1
SKL2-1
SKL3-2
SKL1
Taichung 65
160,119bp
Nipponbare
216,438bp
C9285
333,717bp
Bhadua
333,309bp
SKL3-1
SK2-like1
SKL2-2
SK1
SK2
SKL3-3
10kb
SK2-like2
SKL4-2
Supplementary Figure S2

## Slide 3
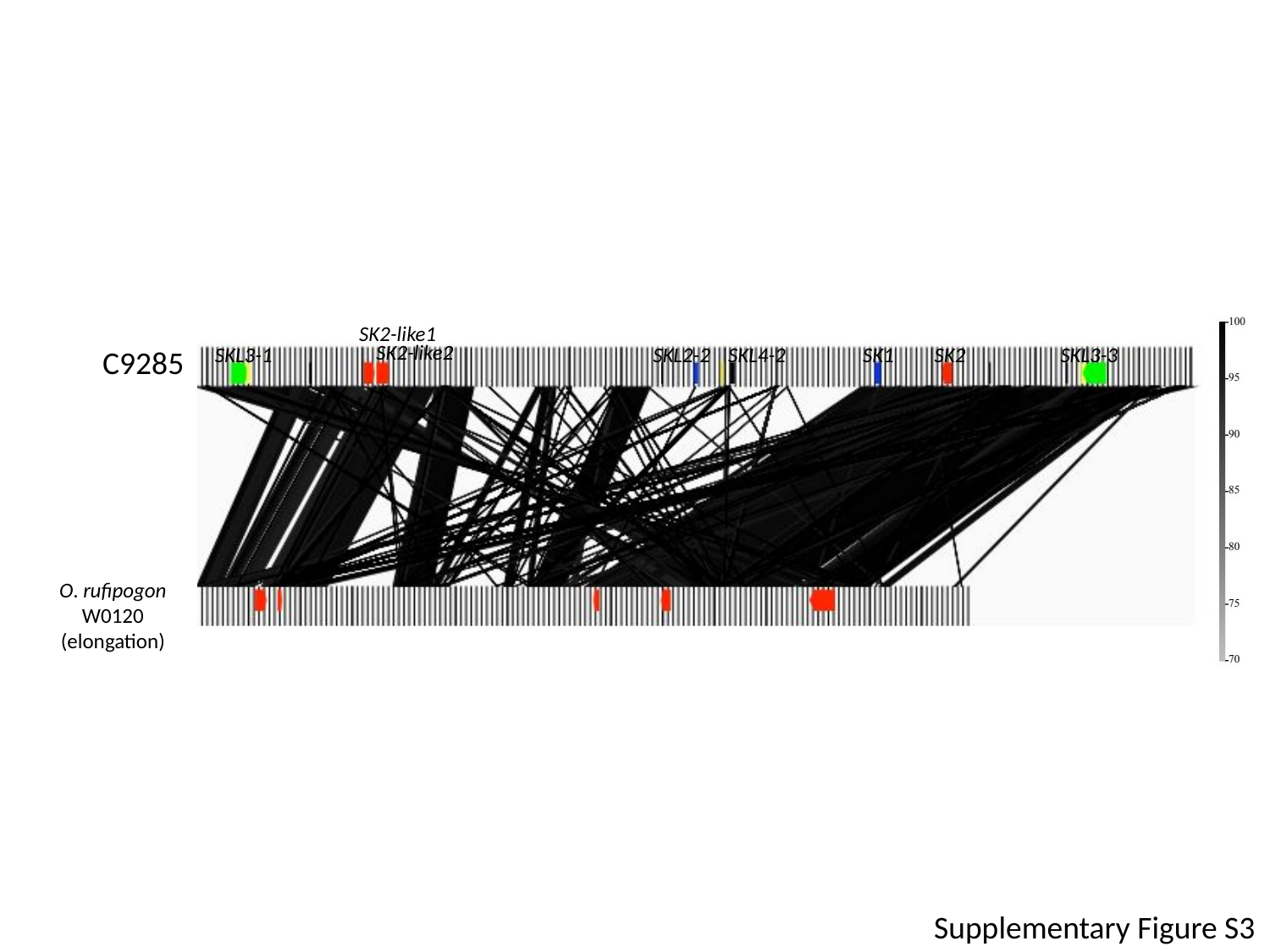

SK2-like1
SK2-like2
SKL3-1
SKL2-2
SKL4-2
SK1
SK2
SKL3-3
C9285
O. rufipogon
W0120
(elongation)
Supplementary Figure S3

## Slide 4
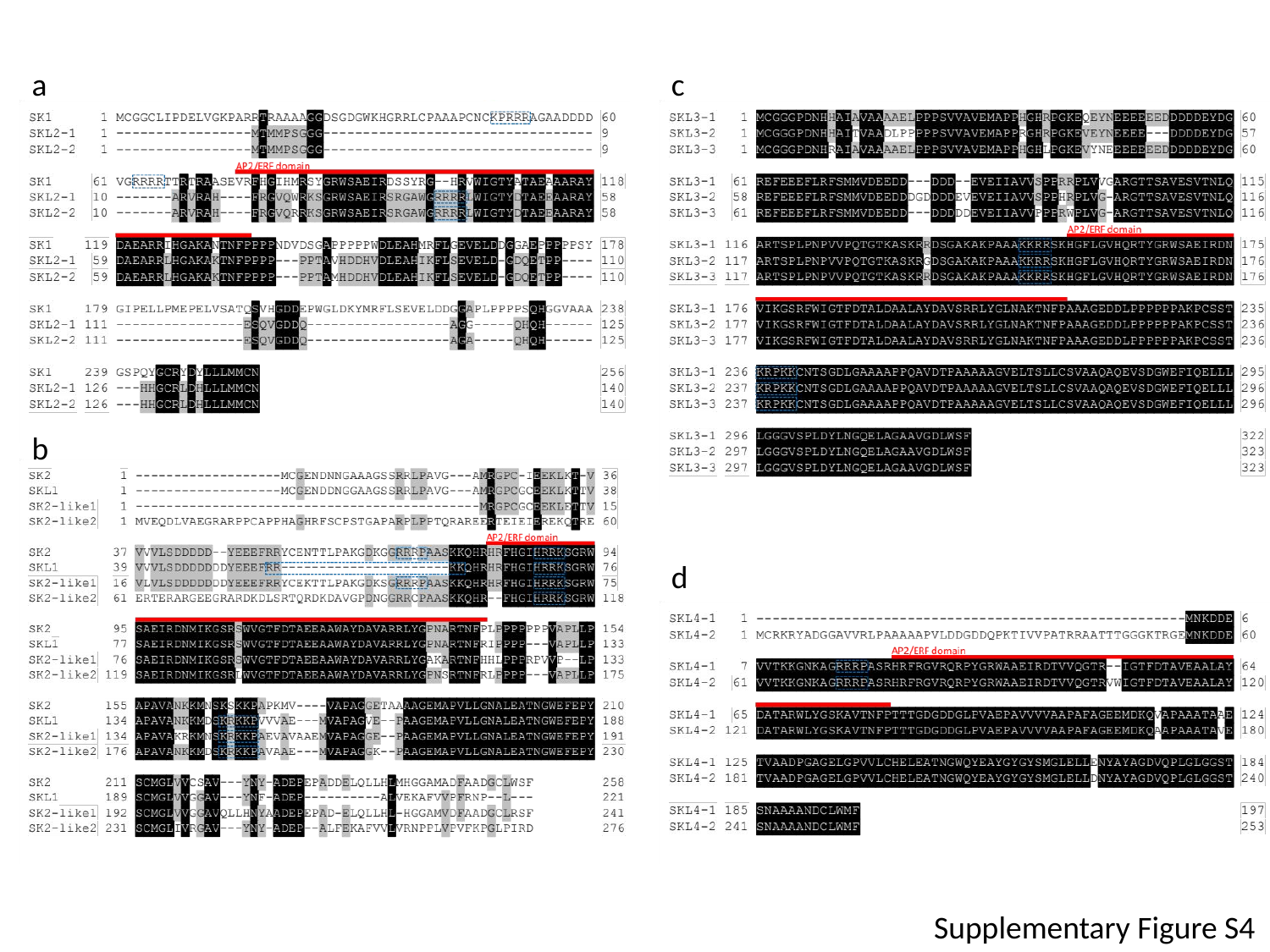

a
c
b
d
Supplementary Figure S4

## Slide 5
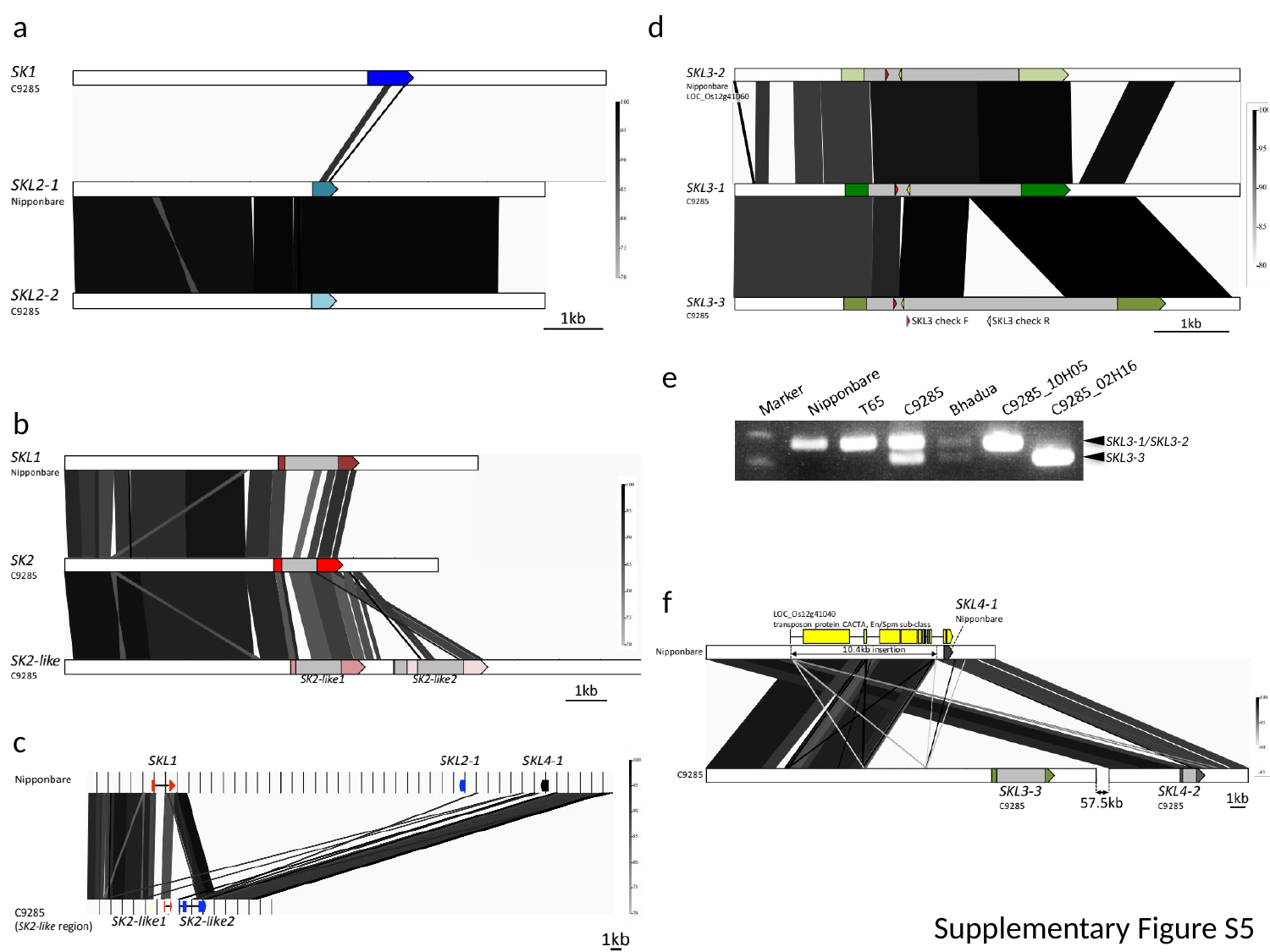

a
d
e
b
SKL3-1/SKL3-2
SKL3-3
f
c
Supplementary Figure S5

## Slide 6
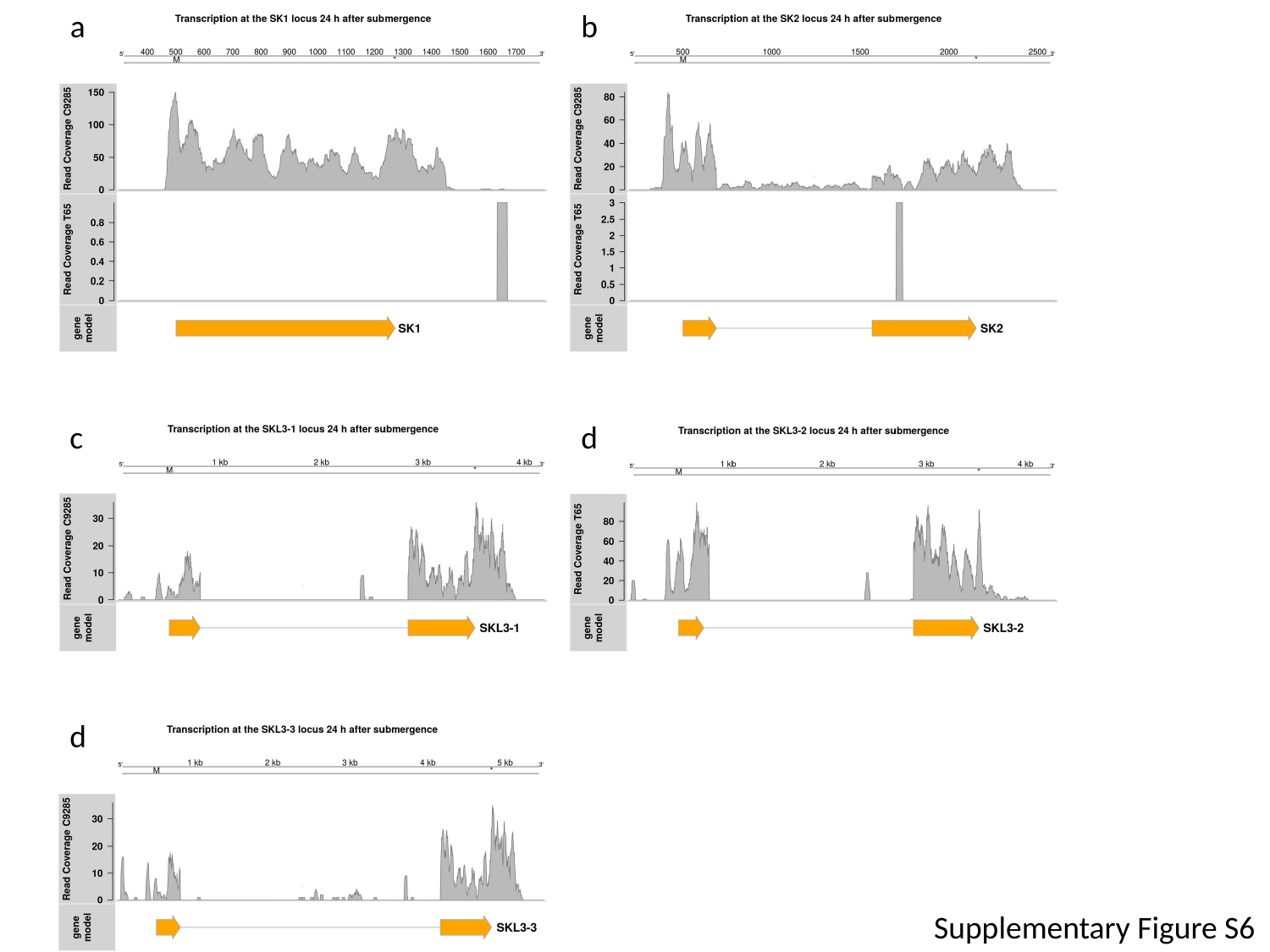

a
b
c
d
d
Supplementary Figure S6

## Slide 7
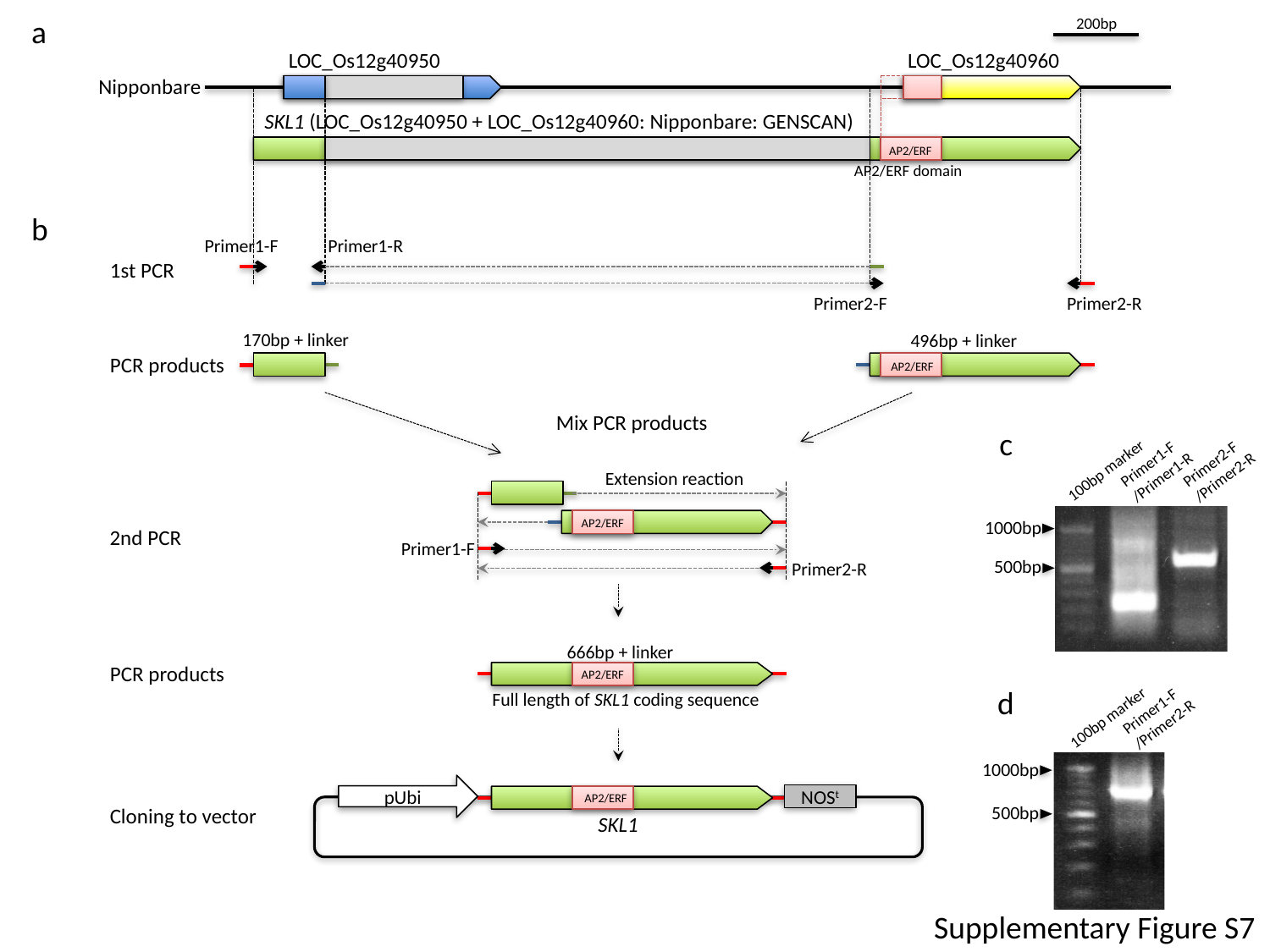

a
200bp
LOC_Os12g40950
LOC_Os12g40960
Nipponbare
SKL1 (LOC_Os12g40950 + LOC_Os12g40960: Nipponbare: GENSCAN)
AP2/ERF
AP2/ERF domain
b
Primer1-R
Primer1-F
1st PCR
Primer2-R
Primer2-F
170bp + linker
496bp + linker
PCR products
AP2/ERF
Mix PCR products
c
Primer1-F
/Primer1-R
Primer2-F
/Primer2-R
1000bp
500bp
100bp marker
Extension reaction
AP2/ERF
2nd PCR
Primer1-F
Primer2-R
666bp + linker
PCR products
AP2/ERF
d
Primer1-F
/Primer2-R
100bp marker
1000bp
500bp
Full length of SKL1 coding sequence
pUbi
AP2/ERF
NOSt
Cloning to vector
SKL1
Supplementary Figure S7
