# Supplementary material for: SNORKEL Genes Relating to Flood Tolerance Were Pseudogenized in Normal Cultivated Rice
Source: Plants (Basel). 2022 Jan 29;11(3):376. doi: 10.3390/plants11030376 (PMC8840289; doi:10.3390/plants11030376)
Supplement: Supplementary file 1 [file plants-11-00376-s001.zip › Supplementary Figures captions (Nagai et al).pdf]

Supplementary Figure S1. Location of ERF family genes on Nipponbare chromosomes. Colored boxes indicate tandem duplicated ERF genes. The gene locus IDs follow [10]. The colored squares next to the gene IDs represents *ERF*s that exist as a cluster, respectively. The gene locations were determined using the chromosome map tool (http://viewer.shigen.info/oryzavw/maptool/MapTool.do). Among the genes listed in Nakano et al. (2006), LOC_Os06g09690 and LOC_Os06g09730 could not be detected with this tool.

Supplementary Figure S2. Comparison of multiple sequences of *SNORKEL* gene regions between normal cultivated rice (Taichung 65 and Nipponbare) and deepwater rice (C9285 and Bhadua).

Supplementary Figure S3. Sequence comparison between deepwater rice (C9285) and wild rice (*O. rufipogon*, W0120).

Supplementary Figure S4. Comparison of protein sequences.
Protein sequences of the (a) SK1, (b) SK2, (c) SKL3, and (d) SK4 types. The amino acid sequences were aligned using ClustalW. The red and blue dashed lines represent the AP2/ERF domain and nuclear localization signal, respectively.

Supplementary Figure S5. Comparison of genomic sequence.
Sequence comparison of the genomic regions of the (a) SK1 type, (b) SK2 type, (c) Nipponbare and SK2-like genes of C9285, (d) SKL3 type, (e) SKL3 alleles, and (f) SKL4 type. The gray region in each gene model indicates an intron.

Supplementary Figure S6. Representative raw RNA-Seq based on transcripts of deepwater rice submerged for 24 hours. Yellow arrows indicate exons.

Supplementary Figure S7. Construction of the plasmid for SKL1 overexpression.
(a) Gene structure of SKL1, as predicted by GENSCAN. LOC_Os12g40960 harbors a truncated form of the AP2/ERF domain in the N-end region, while LOC_Os12g40950 lacks an AP2/ERF domain in its sequence. (b) Cloning the SKL1 CDS. The red region indicates the AP2/ERF domain. Arrows indicate primers. Exons predicted by GENSCAN were amplified in the first PCR step. The second PCR was performed with the PCR products obtained in the first PCR and Primers 1-F and 2-R. The second PCR product was cloned into the vector for overexpression. Electrophoresis of the (c) first and (d) second PCRs.
